# Human-Inspired Eigenmovement Concept Provides Coupling-Free Sensorimotor Control in Humanoid Robot

**DOI:** 10.3389/fnbot.2017.00022

**Published:** 2017-04-25

**Authors:** Alexei V. Alexandrov, Vittorio Lippi, Thomas Mergner, Alexander A. Frolov, Georg Hettich, Dusan Husek

**Affiliations:** ^1^Institute of Higher Nervous Activity and Neurophysiology, Russian Academy of ScienceMoscow, Russia; ^2^Department of Neurology, University Clinics of FreiburgFreiburg, Germany; ^3^Russian National Research Medical UniversityMoscow, Russia; ^4^Institute of Computer Science, Academy of Science of the Czech RepublicPrague, Czechia

**Keywords:** human sensorimotor system, neuromechanics, biorobotics, motor control, eigenmovements

## Abstract

Control of a multi-body system in both robots and humans may face the problem of destabilizing dynamic coupling effects arising between linked body segments. The state of the art solutions in robotics are full state feedback controllers. For human hip-ankle coordination, a more parsimonious and theoretically stable alternative to the robotics solution has been suggested in terms of the Eigenmovement (EM) control. Eigenmovements are kinematic synergies designed to describe the multi DoF system, and its control, with a set of independent, and hence *coupling-free*, scalar equations. This paper investigates whether the EM alternative shows “real-world robustness” against noisy and inaccurate sensors, mechanical non-linearities such as dead zones, and human-like feedback time delays when controlling hip-ankle movements of a balancing humanoid robot. The EM concept and the EM controller are introduced, the robot's dynamics are identified using a biomechanical approach, and robot tests are performed in a human posture control laboratory. The tests show that the EM controller provides stable control of the robot with proactive (“voluntary”) movements and reactive balancing of stance during support surface tilts and translations. Although a preliminary robot-human comparison reveals similarities and differences, we conclude (i) the Eigenmovement concept is a valid candidate when different concepts of human sensorimotor control are considered, and (ii) that human-inspired robot experiments may help to decide in future the choice among the candidates and to improve the design of humanoid robots and robotic rehabilitation devices.

## Introduction

Most human skeletal movements involve several interconnected body segments. Starting from buttressing segments such as the feet when standing, a chain of segments interleaves to the end effector such as the hand in reaching. The joint rotations in such a reaching-while-standing movement occur in a coordinated way, with two aims dictated by physics standing out. A *kinematic* aim is to maintain the center of mass (COM) of all body segments supported by the ankle joints above the base of support, which is the area under and between the feet, in order to maintain balance against external forces acting on the body such as gravity. A *kinetic* aim of movement coordination is to minimize effects of inter-segmental coupling torques. Dysfunction in the matching of timing and torque magnitudes across the chain of segments results in inappropriate compensation for body segment masses and inertia and neural time delays, imposing clinically for the kinematic chain as balance problems (Massion, 1992; Mergner, 2012) and for the kinetic chain as irregular and oscillating movements, a pathological symptom called ataxia that is typically found in cerebellar patients (Bastian, 1997). So far neuroscientists devoted considerable attention to the neural mechanisms underlying human kinematic coordination (Massion, 1992), but paid less attention to the neural mechanisms underlying kinetic coordination, on which this paper focuses.

In the technical domain, where industrial robotic devices are often fixed to the ground, kinematic coordination plays a minor role and the kinetic problem in controlling a chain of serially connected links with coupled dynamics such as a robotic arm can easily be solved. The solution is traditionally done by a full state approach, meaning that feedback and feed forward controls of all joints are computed together in a coordinated way that takes into account a full dynamic model of the arm and solves the inverse dynamics problem. In humanoid robots controlling position of an unstable body posture, the situation is more complex and inter-link force compensation is often performed using servo controllers, one for each joint. If feedback time delays are too large to be fully accounted for by predictive algorithms and if damping of the dynamic coupling effects is insufficient, destabilization of the control may result (Ott et al., 2014, 2016). Also, problems of control stability may arise in humanoid robots with several degrees of freedom (DoF) when the body dynamics are not fully known. Measuring acceleration in each link or a distal link using inertial sensors may help to solve the problem. Also, learning algorithms can be used to produce the needed coordination patterns. Usually reinforcement learning is employed in this context where the desired output is known in terms of performance, but not yet in terms of the needed controller outputs.

In the biological domain, neuroscientists studied for example the electromyographic effects from externally evoked coupling forces in the arm muscles. They observed typical response patterns in muscle activity (Lacquaniti and Soechting, 1986) belonging to the long-latency reflexes, which take into account the current arm configuration (Kurtzer et al., 2009) with a response amplitude scaling that involves the cerebellum (Kurtzer et al., 2013). The underlying neural control mechanisms are still unknown. Theoretically, at least, one could conceive that humans use a neural representation of a full state control. A more parsimonious solution has been suggested in terms of the Eigenmovement (EM) concept (Alexandrov et al., 2001a,b, 2005; Alexandrov and Frolov, 2011). It allows designing the control of the kinematics of the chain in the form of independent SISO (single input, single output) controllers.

Since the EM principle often produces coaction of joint torques in the context of predetermined kinematic synergies, there exists a clear overlap with the important concepts of motor primitives and modular control. Many aspects of motor modularity are discussed by d'Avella et al. (2015) and Flash and Bizzi (2016) including its theoretical and experimental substantiations and robotic applications. According to the modularity concept any movement can be decomposed as a superposition of motor primitives or synergies used as building blocks in a modular control architecture. Each module imposes as a specific pattern of motor activity in terms of kinematic, kinetic or EMG synergy. The specificity of an EM mechanism in this context is that it solves the problem of the dynamic coupling as the basis for controlling each kinematic synergy independently from the others. Although the independent control was demonstrated experimentally so far mostly in relatively simple movements we conceive that the EM concept can be extended to the wide class of multi-joint movements (see Discussion, also for robotic implementations).

Development of the EM concept started from biomechanically describing human hip-knee-ankle coordination during trunk bending as movements along eigenvectors of the motion equation (Alexandrov et al., 2001a,b). After showing that the contribution from the knee joints to this coordination tends to be negligible, the approach was restricted to hip-ankle coordination, and it was shown that the concept is applicable to independently controlled feed-forward and feedback situations (Alexandrov and Frolov, 2011) and that postural reactions to external perturbations can be formalized using a PD (proportional, derivative) control with time delays in the feedback loops (Alexandrov et al., 2005). In their studies, the authors considered the possibility that humans use EM controllers in some neurally implemented form. However, similar as with other concepts of human sensorimotor control, the evidence is indirect and still rather limited as long as analogies are drawn mainly from mathematical calculations or computer simulations, while biological constrains such as neural feedback time delays have experimentally not been considered in face of “real world” challenges such as noisy and inaccurate sensors and non-linearities from computational and mechanical “dead zones” and friction, backlashes, etc. Such limitations may constrain also the potential use of the EM concept in humanoid robots and robotic rehabilitation devices.

**Figure 1:**
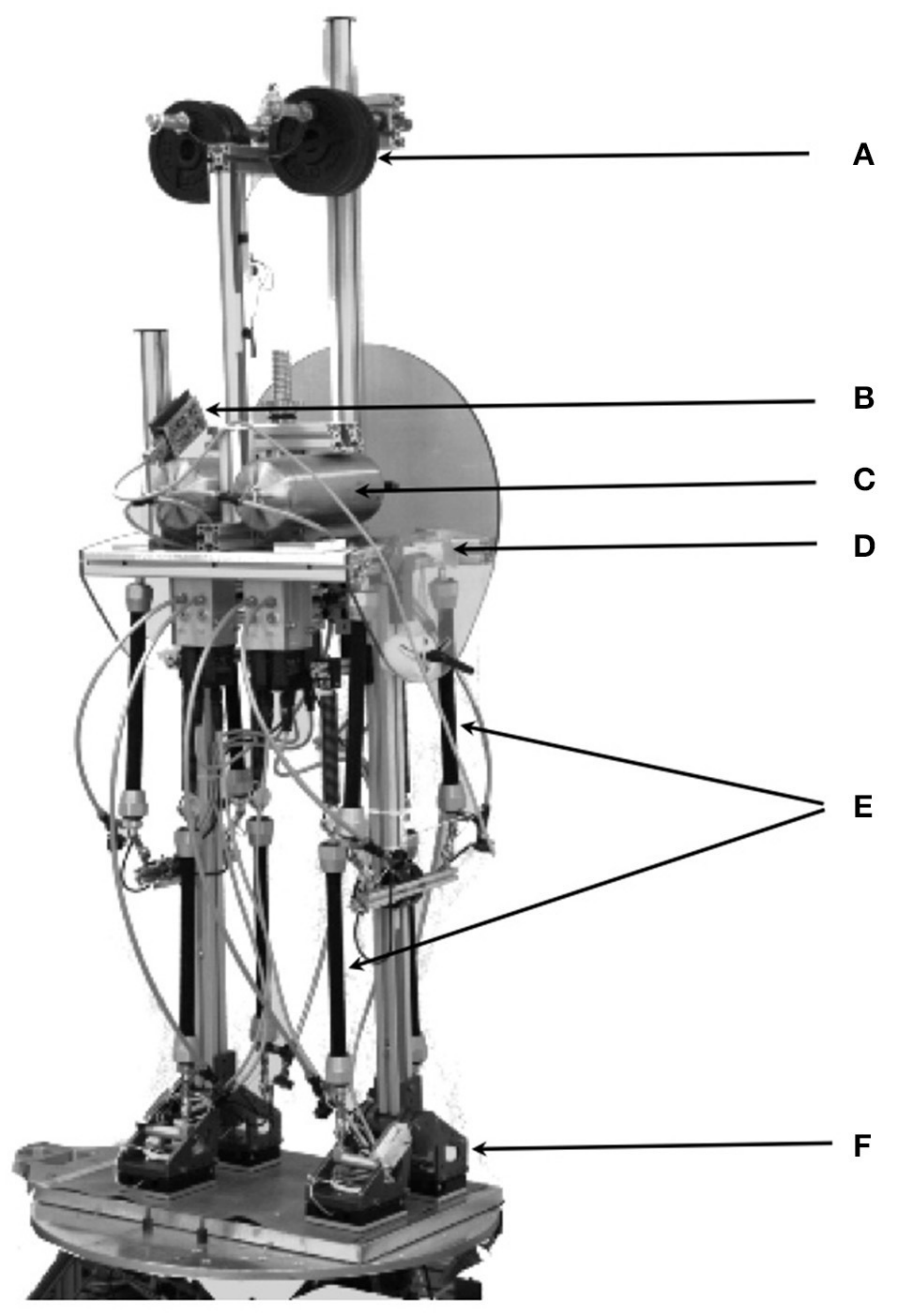
**Postural humanoid robot PostuRob II standing freely on a 6 DOFs motion platform**. (A) weights for human-like weight distribution; (B), artificial vestibular system; (C), pneumatic system; (D), hip joint (joint angle and torque sensors); (E), artificial pneumatic “muscles”; (F), ankle joint (joint angle and torque sensors). (See Appendix 1 in Supplementary Material for details)

With these reservations in mind, this paper investigates whether the EM concept is able to control a humanoid robot that shows human anthropometrics and is equipped with human-inspired sensors and actuators. The robot used, PostuRob II (Figure 1), served already before in experiments that tested a human-derived control concept as robotic implementation (Hettich et al., 2014). Similarly, it is currently used in modified form in another neurorobotics study for the overarching goal to experimentally evaluate the “real-world robustness” of human-inspired control concepts and to obtain back from the robot experiments inspirations for the human sensorimotor research.

The following sections describe first the hip-ankle biomechanics model and the EM controller and its operational capabilities. The subsequent sections describe identification of the robot's specific transfer characteristics by estimating its inertial, gravitational and geometric parameters and the properties of the transformation from joint torque commands at the controller output to the actual torques that were experimentally observed at the joints (which in autonomous systems may be achieved by learning). Then, experimental results from testing the robot in a human posture control laboratory are described, including preliminary comparisons with human data, followed by Discussion. Details of the mathematical concepts, the robot, and experimental procedures are given in Appendices.

## Materials and methods

### Eigenmovement (EM) concept and biomechanical hip-ankle model

**Figure 2:**
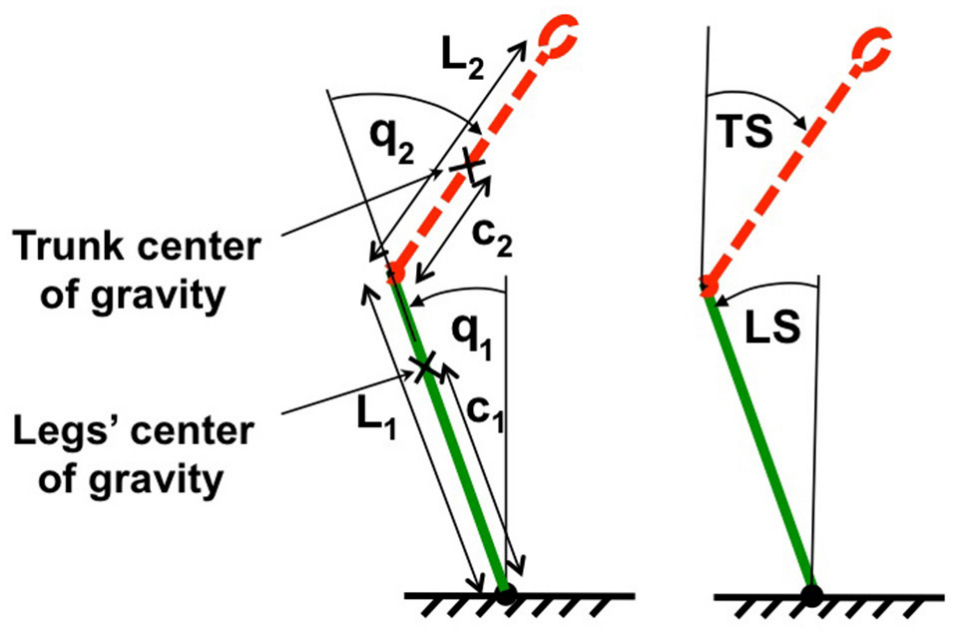
**Two-link biomechanical model of the humanoid robot**. The trunk is represented by red dotted line and the legs are represented by a green solid line. L1, L2, c1, and c2 are lengths and locations of centers of mass for legs and trunk respectively. On the left body position is expressed in terms of joint coordinates (ankle and hip angles, q_1_ and q_2_), on the right in terms of space coordinates *trunk in space* (TS) and *leg in space* (LS) relative to the gravitational vertical.

Humanoid sagittal movements around hip and ankle joints in the vicinity of vertical body position (Figure 2) are described by

(1) $$B_{0}\ddot{q}-G_{0}q=\tau^{\text{con}}$$

where ***q*** is the vector of hip and ankle joint angles, ***B***_0_ and ***G***_0_ are the inertial and gravity matrices, and τ^con^ is the vector of joint control torques. The coefficients of ***B***_0_ and ***G***_0_ are calculated via the length-mass parameters of Posturob II, as described in Appendix 1 in supplementary Material.

Each EM is in the linear approach the movement along one eigenvector *w*_*i*_ that, by definition, satisfies equation

(2) $$\begin{array}{r} B_{0}w_{i}=\lambda_{i}G_{0}w_{i},\;\left(i\;=\;1,\;2\right) \end{array}$$

where λ_*i*_ is the corresponding eigenvalue (Alexandrov et al., 2001a). The vector ξ of the time courses of the two EMs is obtained by transforming the vector ***q*** by inversion of equation.

(3) $$\begin{array}{r} q\left(t\right)=W\xi \left(t\right) \end{array}$$

where the two columns of matrix ***W*** are the eigenvectors ***w***_*i*_, (*i* = 1,2). According to Equations (2) and (3), the dynamic equation (1) takes the following form in terms of EMs.

(4) $$\begin{array}{r} \Lambda \ddot{\xi }-\xi =\eta^{con} \end{array}$$

where Λ is a diagonal matrix with eigenvalues λ_*i*_, and

(5) $$\begin{array}{r} \eta^{con}=U\tau^{con},\;\text{U}={\left(G_{0}W\right)}^{-1} \end{array}$$

The two columns ***u***_*i*_ (*i* = 1,2) of matrix ***U*** in Equation (5) are the vectors, whose components define the contributions of ankle and hip joint torques to the EM dynamics.

### EM PD-controller

As shown previously (Kuo, 1995; Welch and Ting, 2008; Frolov et al., 2000, 2006), the joint torques τ^con^, which generates the desired body movement, can be implemented as a PD-controller with time delay Δ*t* in the form

(6) $$\begin{array}{l} \tau^{con}(t)\;=\;-G_{0}q(t-\Delta t)+S(q^{d}(t-\Delta t)-q(t-\Delta t))\\ \;-V\dot{q}(t-\Delta t) \end{array}$$

where ***q***^*d*^ and ***q*** define the time course of the desired and actual changes in joint angles, while ***S*** and ***V*** represent “stiffness” and “viscosity” matrices whose elements define the gain coefficients in the feedback loop.

In EMs, Equation (6) takes the form:

(7) $$\begin{array}{rcl} \eta^{con}\left(t\right) & = & -\xi \left(t-\Delta t\right)+S^{eig}\left(\xi^{d}\left(t-\Delta t\right)-\xi \left(t-\Delta t\right)\right)\\ -V^{eig}\dot{\xi }\left(t-\Delta t\right) \end{array}$$

where

(8) $$\begin{array}{r} S^{eig}\;=\;{\left(G_{0}W\right)}^{-1}\mathrm{SW},\;V^{eig}\;=\;{\left(G_{0}W\right)}^{-1}\mathrm{VW} \end{array}$$

Independent control of each of the two EM means that matrices ***S***^*eig*^ and ***V***^*eig*^ are diagonal, so that the vector Equation (1) in terms of joint angles and torques splits into two scalar equations in terms of EMs (Alexandrov et al., 2001a, 2005; Alexandrov and Frolov, 2011), each equivalent to a PD-control of a single-link inverted pendulum:

(9) $$\begin{array}{rcl} \lambda_{i}\xi_{i}\left(t\right)-\xi_{i}\left(t\right) & = & -\xi_{i}\left(t-\Delta t\right)+{\text{S}}_{i}^{eig}\left[\xi_{i}^{d}\left(t-\Delta t\right)-\xi_{i}\left(t-\Delta t\right)\right]\\ -V_{i}^{eig}{\dot{\xi }}_{i}\left(t-\Delta t\right) \end{array}$$

where ***S***_*i*_^*eig*^ and ***V***_*i*_^*eig*^ are the diagonal elements of matrices ***S***^*eig*^ and ***V***^*eig*^, respectively (λ = *J/mgh*; *J*, moment of inertia relative to pendulum axis of rotation; *m*, pendulum mass and *h* its altitude; *g*, gravitational acceleration).

The inverse transformation of Equation (8) gives the stiffness and viscosity matrices ***S*** and ***V*** in Equation (6) in terms of joint angles:

(10) $$\begin{array}{r} S=\;G_{0}WS^{eig}W^{-1},\;V=\;G_{0}WV^{eig}W^{-1}. \end{array}$$

When matrices ***S***^*eig*^ and ***V***^*eig*^ are diagonal, then matrices ***S*** and ***V*** are symmetrical, but not diagonal (Alexandrov et al., 2005). Therefore, the PD-control in terms of joint angles needs to take into account not only the kinematics and dynamics of a given joint, but also those of all other joints. The number of feedback control parameters in this kind of control, referred to as “full-state feedback control” (Barin, 1989; Park et al., 2004), is equal to the number of elements in the stiffness and viscosity matrices ***S*** and ***V***. In the EM approach, notably, the number of feedback control parameters is reduced to the number of diagonal coefficients in the matrices ***S***^*eig*^ and ***V***^*eig*^.

**Figure 3:**
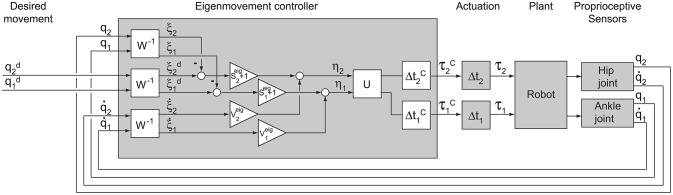
**Scheme of EM control**. Shown are the EM controller, the actuation, the plant and the sensors. The PD-controller transforms desired and sensory variables ***q*** into EM kinematic variables ξ (boxes *W*^−1^) and then into EM dynamic variables η^con^ (boxes S^eig^ + 1 and V^eig^) which are in turn transformed into output joint torques τ^*C*^ (box U) with controller time delays (boxes Δ*t*^*C*^). Torque τ^*C*^ becomes effective at the robot segment after an actuation time delay (boxes Δt). Note, that in boxes S^eig^ + 1 the unit is added to S^eig^ in order to take into account the first “gravity” term in the right side of Equation (7). Matrices ***S***^eig^, ***V***^eig^, ***W*** and ***U*** are defined above.

The EM PD-controller is shown schematically in Figure 3. The time delays outside the controller represent time delays Δ*t*_1_ and Δ*t*_2_ between controller commands τ^*C*^ and actual torque τ applied to the robot segments, which are mainly induced by the generation of the torques in the robot. These “actuation” delays were intentionally equalized in the present study inside the PD-controller by adding delays of Δ$t_{1}^{C}$ and Δ$t_{2}^{C}$ to the respective joints (Figure 3) such that the total time delays Δ*t*_*A*_ and Δ*t*_*H*_ of the transformation for ankle and hip joint torques respectively were equal and amounted to Δ*t*_*A*_ = Δ*t*_1_ + Δ$t_{1}^{C}$ = Δ*t*_*H*_ = Δ*t*_2_ + Δ$t_{2}^{C}$ = Δ*t*.

For the robot experiments (see below), the controller has been implemented as a program in Simulink, which allows controlling the robot in real time. As to the controller inputs, the joint angles *q* give the desired body position in terms of joint angles with respect to each other and the support surface. This is by itself not sufficient to balance in the general case in which the support surface is not a stable reference. The Posturob platform integrates a human inspired vestibular system, mechanically fixed to the upper body and providing the trunk orientation in space (Mergner et al., 2009). Using the vestibular information, the control can be generalized to the condition of support surface tilt in space. In particular, in experiments with the robot standing on moving platform, the information of leg-in-space angle was calculated with help of the vestibular sensor and used as input signal *q*_1_ for the leg segment control, and the joint angle signal from the hip joint sensor was used for the hip control. With stable platform, angle and vestibular sensor signals were combined for each joint to improve the signal to noise ratio.

### Theoretical analysis of control stability

The independent control for each EM allows the analysis of whole body control stability by two separate analyses of each EM's stability. The stability of each EM is defined by the roots μ of the secular equation of Equation (9):

(11) $$\begin{array}{r} \mu^{2}\;\lambda -1+\left({\text{S}}^{\text{eig}}+1\right){\text{e}}^{-\mu \Delta \text{t}}+\mu {\text{V}}^{\text{eig}}{\text{e}}^{-\mu \Delta \text{t}}=0. \end{array}$$

When in equation (9) Δt > 0, Equation (11) has an infinite number of complex roots μ = α + *i*ω, where α and ω are the real and imaginary parts of the root and *i* is the imaginary unit (Alexandrov et al., 2005). The solution of equation (9) is stable if the real part α of all the roots of equation (11) is negative. The maximum value of the real part of all the roots of the secular equation is called *Lyapunov index*. Thus, the solution of equation (9) is stable if its Lyapunov index α < *0*. The Lyapunov index defines the characteristic time Δ*t*_*chr*_ = |α|^−1^ of the complex system response to the external perturbation.

**Figure 4:**
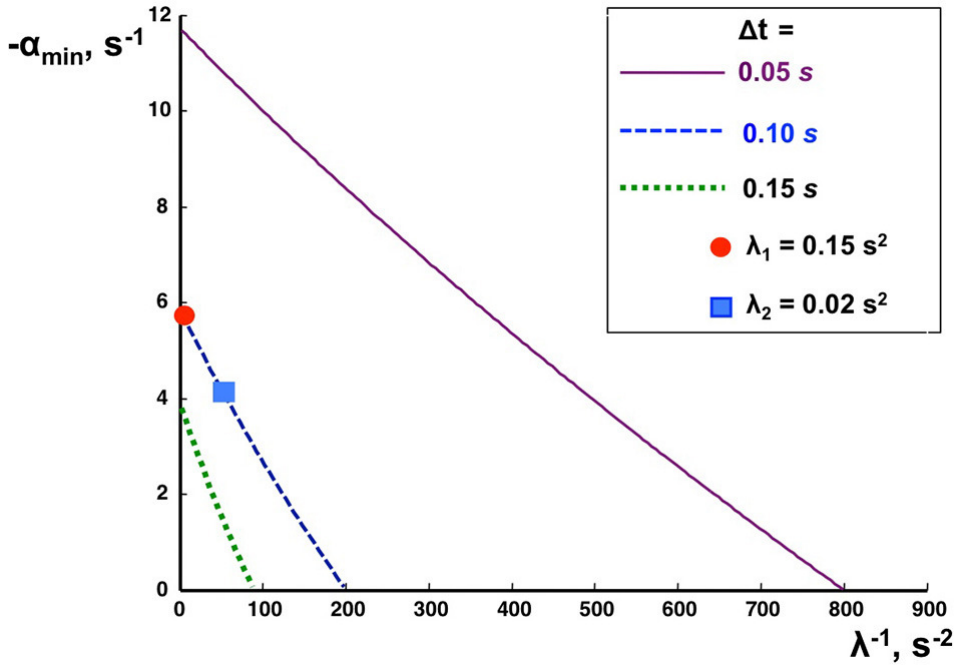
**Effects of feedback delay time on PD-control stability**. The negative Lyapunov index α_*min*_ (ordinate), is plotted as a function of the inversed index of inertia λ (abscissa) for different delay times Δt as indicated.

The minimization of the Lyapunov index for each EM was used as a criterion for optimizing the PD-controller parameters. The optimum parameters were obtained according to a method based on calculations of the ranges in the space of *S*^eig^ and *V*^eig^ in which the Lyapunov index does not exceed given values α (Appendix 2 in Supplementary Material). The main results are shown in Figure 4. It shows the minimal Lyapunov index α_min_ which can be achieved for a given λ and Δ*t*. The values of *S*^eig^ and *V*^eig^ which provide α_min_ were treated as optimal. For each delay Δ*t* there exists some critical value λ_crit_ at which α_min_ becomes zero. If λ < λ_crit_, no range space of stability for the given feedback loop delay exists, meaning that the PD-controller does not provide stable control of this dynamic system. Thus, stable PD-control is impossible if feedback loop delay Δ*t* > Δ*t*_max_ for a given λ or if λ < λ_crit_ for a given Δ*t*.

### Experimental setup

The humanoid robot PostuRob II (Figure 1) comprises trunk, legs and feet segments interconnected by the hip and ankle joints. Signals from mechatronic vestibular and joint angle sensors are real-time inputs to a PC. The implemented control system controls artificial pneumatic “muscles” (FESTO AG &Co.KG, Esslingen, Germany; Typ MAS20), which generate a desired torque in the hip and ankle joints. The EM control model was executed as a compiled Simulink model (Real-Time Windows Target, The Math Works Inc., Natick, USA). In the presented experiments, the robot was standing freely on firm support, a 6 DoF motion platform (Mergner et al., 2003), and performed active sinusoidal trunk and leg movements in the sagittal plane with different frequencies and amplitudes, reactive postural responses to external disturbances such as support surface rotation or translation in the sagittal plane. During the experiments, performed in a human posture control laboratory, sensory signals of joint angles and joint angular velocities as well as desired joint angle signals and actual joint torques were recorded with an acquisition rate of 200 Hz (further details in Appendix 1 in Supplementary Material).

In the first series, the robot's characteristics were evaluated in terms of (*a*) its *inertial and gravity matrices **B***_0_ *and **G***_0_, (*b*) the “*actuation” time delays* Δ*t*_1_ and Δ*t*_2_ in the transformation of *joint torque commands to effective torques* at the robot's joints, (*c*) the optimal *PD-controller parameters*, and (*d*) the *dynamic response characteristics* of the robot and *(e) Experimental transfer functions and* dynamic response of the controlled system. For *a*, preliminary (theoretically optimal) parameters of the PD-controller were calculated based on the model of the two-linked rigid rods described above, and for *b* the time delays Δ*t*_1_ and Δ*t*_2_ for the transformation of torque commands to the effective joint torques were preliminarily estimated to be 100 ms. This allowed for stable PostuRob II movements within the tested conditions and, at a later step (comparing the theoretical with the measured transfer functions), to finally calculate optimal parameters of the PD-controller on the basis of the experimentally obtained matrices ***B***_0_ and ***G***_0_.

#### Experimental stimuli

The commanded joint angles in this approach, $q_{1}^{d}\left(t\right)$ for the ankle joint and $q_{2}^{d}\left(t\right)$ for the hip joint in Equation (1), had synchronous sinusoidal time courses with seven different frequencies *f*: 0.05, 0.1, 0.2, 0.4, 0.6, 0.8 and 1.0 Hz. Five pairs of amplitudes *A*_1_ and *A*_2_ of desired signals $q_{1}^{d}\left(t\right)=A_{1}\text{sin}\left(2\pi ft\right)$ and $q_{2}^{d}\left(t\right)=A_{2}\text{sin}\left(2\pi ft\right)$ were tested for each frequency: (*A*_1_, *A*_2_) = (0, 10°), (−2.5°, 7.5°), (−5°, 5°), (−4.5°, 1.5°), and (3°, 0). With these amplitudes, the zero moment point position remained inside the support area defined by the area comprised by the feet.

#### Evaluation of the robot's inertial and gravity matrices

The evaluation was performed by integration of Equation (1) with the help of the experimentally recorded joint torques and a given set of elements for matrices ***B***_0_ and ***G***_0_ (compare Kuo, 1995; Alexandrov and Frolov, 2011). The elements of these matrices, which minimize the error between the joint angles obtained by the numerical integration and the experimental joint angles, were taken for estimating the robot's characteristics. Their search was performed by the gradient descent method given in the MATLAB software. Parameters presented in Appendix 1 in Supplementary Material were taken as the initial ones. The search terminated on the step when the tolerance function decreased less than by 10^−4^ as compared with the previous step.

#### Evaluation of time delays in transformation from torque commands to effective joint torques

The transformation from the joint torque commands at the PD-controller output to the experimentally obtained joint torques is realized in PostuRob II independently for the hip joint and the ankle joint. Crosstalk between the joint torques is negligible and the transformation is performed in each joint separately with different time delays (boxes Δ*t*_1_, Δ*t*_2_ in Figure 2). The matrix ***F***^ττ^ describes the transfer function of the desired torque commands to the effective torques at the robot segments by

(12) $$\begin{array}{r} F^{\tau \tau }=\;\left(\begin{array}{cc} e^{-i\omega \Delta t_{1}} & 0\\ 0 & e^{-i\omega \Delta t_{2}} \end{array}\right) \end{array}$$

where ω = 2π*f*, *f* is the frequency of the sinusoidal robot movement, *i* is the imaginary unit, and Δ*t*_1_, Δ*t*_2_ are the delays in the transformation of torque commands to the torques in the ankle and hip joints, respectively. The delays Δ*t*_1_ and Δ*t*_2_ that provided the best fit with the experimental transfer function were then taken for the subsequent estimations.

## Results

The experiments with Posturob II were performed on a motion platform in a human posture control laboratory. They comprised in addition to voluntary movement tests also tests of balancing biped stance during external disturbances (details in Appendix 1 in Supplementary Material). We refrained from adjusting the above control parameters to specific experimental conditions even when this was associated with particular technical insufficiencies such as an increased static friction. *Proactive lean movement* and *reactive postural lean responses* were tested. Both could be performed either in space coordinates using the artificial vestibular sensor (change in trunk-space angle, TS, or leg-space angle, LS) or in proprioceptive coordinates (change in trunk-leg angle, TL, or leg-foot angle, LF).

### Evaluation of the robot's inertial and gravity matrices

The elements of matrices ***B***_0_ and ***G***_0_ obtained experimentally amounted to ***B***_011_ = 65.01 N m s^2^ rad^−1^, ***B***_012_ = ***B***_021_ = 10.09 N m s^2^ rad^−1^, ***G***_011_ = 460.01 N m rad^−1^, ***G***_012_ = ***G***_021_ = ***G***_022_ = 103.02 N m rad^−1^ (these values replaced in the following the initial values given in Appendix 1 in supplementary Material). The experimental transfer function ${\text{F}}_{e}^{q\tau }$ was calculated according to Appendix 3 in Supplementary Material for the case that the two components of the signal ***X***(t) are the joint angles and the two components of the signal ***Y***(t) are the corresponding joint torques recorded during the above 35 described cyclic movements of PostuRob II. The theoretical transfer function ${\text{F}}_{t}^{q\tau }$ was calculated according to equation

(13) $$\begin{array}{r} F_{t}^{q\tau }=-\omega B_{0}-D_{0} \end{array}$$

using the inertial and gravity matrices **B**_0_ and **G**_0_ obtained experimentally. The root-mean-square error of the mismatch between ${\text{F}}_{t}^{q\tau }$ and ${\text{F}}_{e}^{q\tau }$ amounted to 9.9%.

### Evaluation of delays in torque actuators

**Figure 5:**
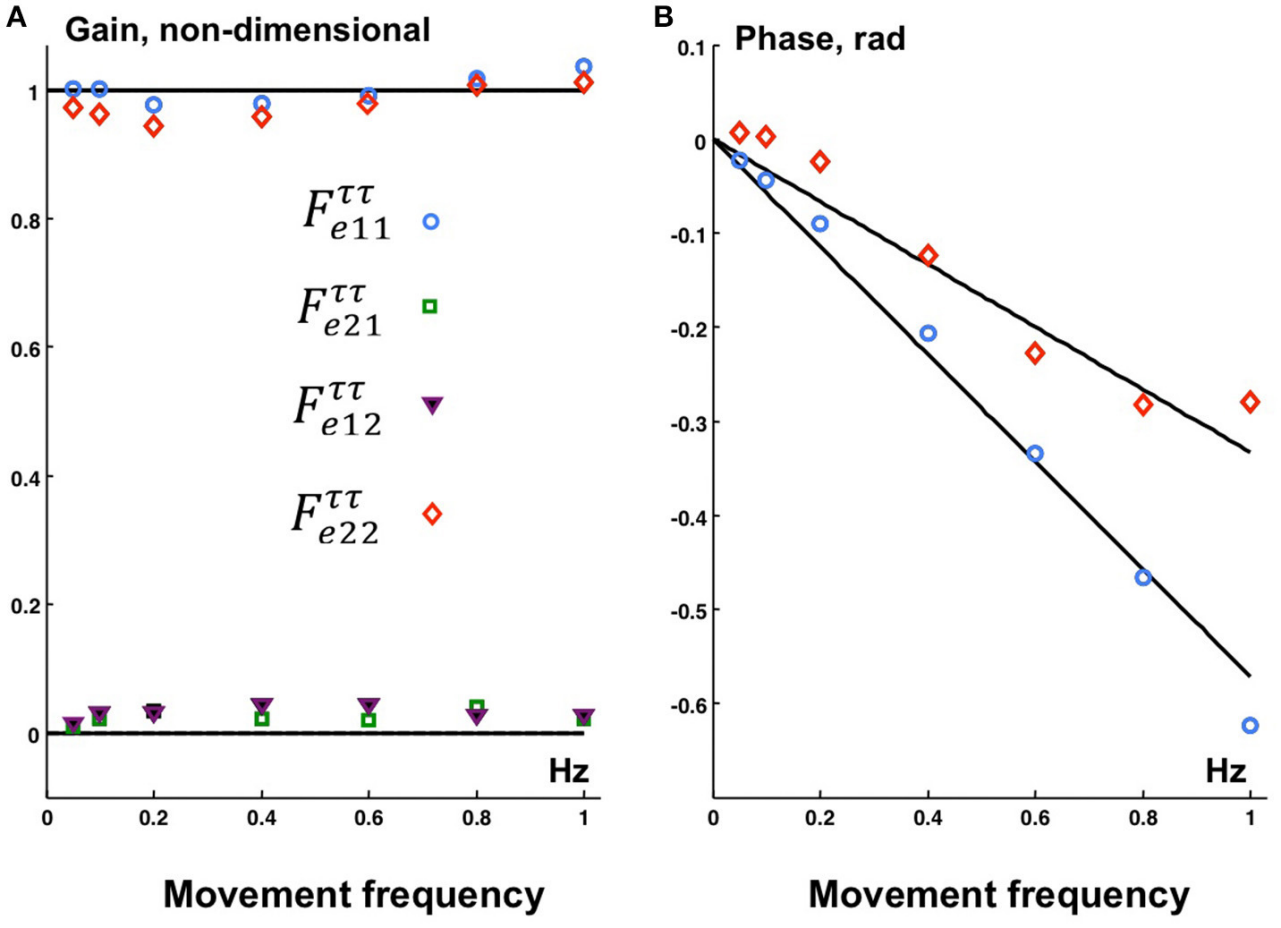
**The gain (A)** and phase **(B)** characteristics of *theoretical* transfer function $F_{t}^{\tau \tau }$ (curves) calculated according to (13) with joint torque delays τ_1_ = 0.091 s (ankle) and τ_2_ = 0.053 s (hip) and *experimental* $F_{e}^{\tau \tau }$ (markers) for the transformation of the *controller output joint torques to the actual joint torques*. Experimental data points: ° $F_{e11}^{\tau \tau }$, □ $F_{e21}^{\tau \tau }$, ▾ $F_{e12}^{\tau \tau }$, ♢ $F_{e22}^{\tau \tau }$.

Figure 5 shows the experimentally obtained transfer function ${\text{F}}_{e}^{\tau \tau }$ and the theoretical transfer function ${\text{F}}_{t}^{\tau \tau }$ for the transformation of the torque commands to the joint torques. The function ${\text{F}}_{t}^{\tau \tau }$ was calculated according to Equation (12) and ${\text{F}}_{e}^{\tau \tau }$ according to Appendix 3 in Supplementary Material. The two components of the signal ***X***(t) are the control signals $\tau_{1}^{C}$ and $\tau_{2}^{C}$ at the PD-controller output (Figure 3) for the ankle and hip joints respectively, and the two components of the signal ***Y***(t) are the corresponding experimental recordings of the joint torques. The “actuation delays” that provided the minimum root-mean-square error between the ${\text{F}}_{e}^{\tau \tau }$ and ${\text{F}}_{t}^{\tau \tau }$ amounted to Δ*t*_1_ = 0.091 s (ankle) and Δ*t*_2_ = 0.053 s (hip). The minimum root-mean-square error amounted to 5.1%. Note that the off-diagonal elements of the experimental transformation matrix ${\text{F}}_{e}^{\tau \tau }$ are small compared to the diagonal elements, indicating very small crosstalk torques between the joints.

### Readjustments of the parameters for the EM PD-controller

The solutions of Equations (2) and (5) for the experimentally obtained matrices ***B***_0_ and ***G***_0_ give the following eigenvalues λ_*i*_, eigenvectors *w*_*i*_, and vectors *u*_*i*_ (*i* = 1, 2) defined in Appendix 1 in Supplementary Material:

(14) $$\begin{array}{r} \lambda_{1}=0.15\;{\text{s}}^{2};\;w_{1}=\;\left(\begin{array}{c} -0.89\\ -0.46 \end{array}\right);\;u_{1}=\;\left(\begin{array}{c} 455.6\\ 138.9 \end{array}\right)\\ \lambda_{2}=0.02\;{\text{s}}^{2};\;w_{2}=\;\left(\begin{array}{c} -0.29\\ -0.96 \end{array}\right);\;u_{2}=\;\left(\begin{array}{c} 35.6\\ -68.5 \end{array}\right) \end{array}$$

The actuation delays Δ*t*_1_ = 0.091 s and Δ*t*_2_ = 0.053 s were intentionally equalized by adding delays of $\tau_{1}^{\text{C}}$ = 0.009 s and $\tau_{2}^{\text{C}}$ = 0.047 s to the respective joint inside the PD-controller (Figure 3). As a result, the total delays for both ankle and hip joint torques amounted to Δ*t* = 0.1 s. The two markers in Figure 4 located on the dashed curve for Δ*t* = 0.1 s and the two eigenvalues λ_1_ and λ_2_ of PostuRob II indicate that the Lyapunov indexes in the experimental movements amounted to α_*min*1_ = −5.6 s^−1^ for the first EM and α_*min*2_ = −4.1 s^−1^ for the second EM.

The following optimal values of stiffness $S_{opt}^{eig}$ and viscosity $V_{opt}^{eig}$ in the PD-controller were calculated according to Appendix 2 in Supplementary Material for the obtained values of λ_1_, λ_2_, and Δ*t* = 0.1 s:

(15) $$\begin{array}{r} S_{opt1}^{eig}=1.04;\;{\text{V}}_{opt1}^{eig}=0.73\;s;\\ S_{opt2}^{eig}=0.06;\;{\text{V}}_{opt2}^{eig}=0.15\;s. \end{array}$$

To obtain the estimate of the transfer function *F*^*qq*^ from desired to actual kinematics, cyclic movements of PostuRob II were recorded using the optimal parameters obtained so far. These recordings were used to calculate an experimental transfer function from desired to actual kinematics (Appendix 3 in Supplementary Material) and were compared to a theoretical transfer function (Appendix 4 in Supplementary Material).

**Figure 6:**
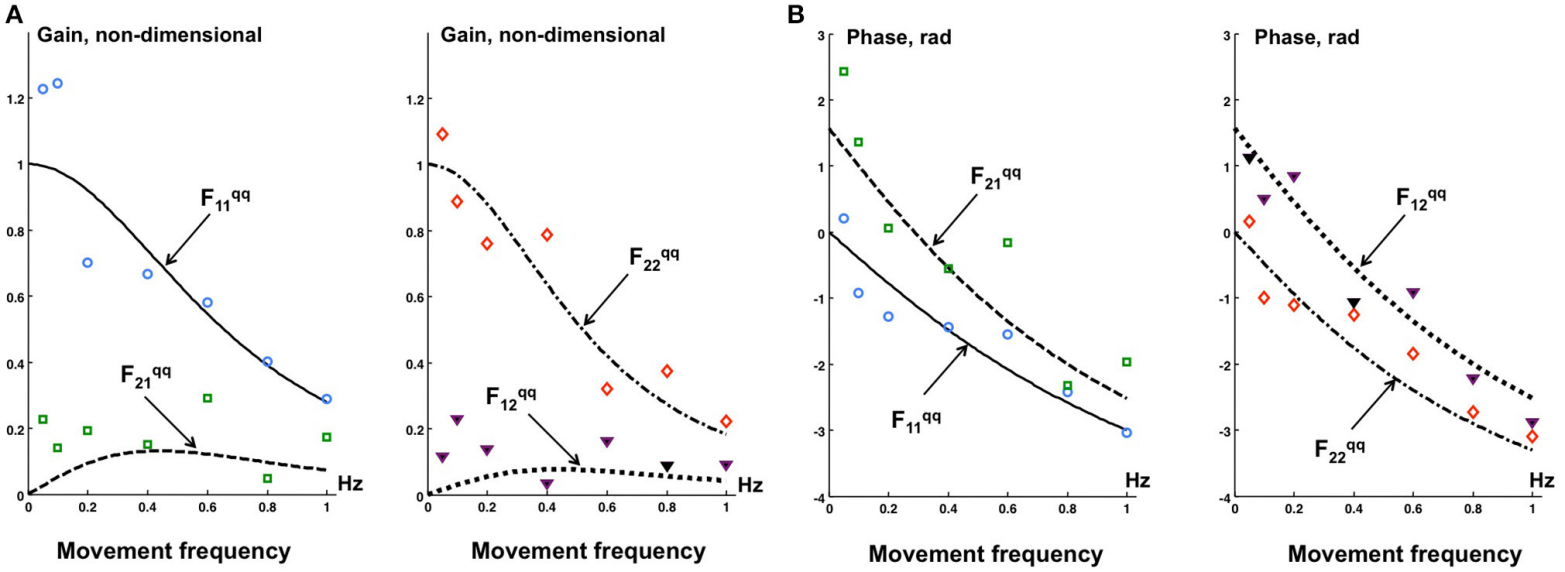
**The gain (A)** and phase **(B)** characteristics of the theoretical $F_{t}^{qq}$ (curves) and experimental $F_{e}^{qq}$ (data points) transfer functions of the desired kinematics to the actual kinematics in terms of joint angles q. Experimental points: ° - $F_{e11}^{qq}$, □ $F_{e21}^{qq}$, ▾ $F_{e12}^{qq}$, ♢ $F_{e22}^{qq}$.

Figure 6 shows the gain (Figure 6A) and phase (Figure 6B) characteristics of the transfer function *F*^*qq*^ from the desired kinematics to the actual kinematics in terms of joint angles. The experimentally obtained values ${\text{F}}_{e}^{qq}$ calculated according to Appendix 3 in Supplementary Material are compared with the theoretical ones ${\text{F}}_{t}^{qq}$ calculated according to Appendix 4 in Supplementary Material.

The off-diagonal elements $F_{12}^{qq}$ and $F_{21}^{qq}$ of the transfer function ***F***^*qq*^ are theoretically and experimentally non-zero. However, the off-diagonal elements are small as compared with the diagonal elements $F_{11}^{qq}$ and $F_{22}^{qq}$. In general, the experimental data points in Figure 6 qualitatively correspond to the theoretical results (curves), this despite some data scatter.

### Proactive movements of the robot

**Figure 7:**
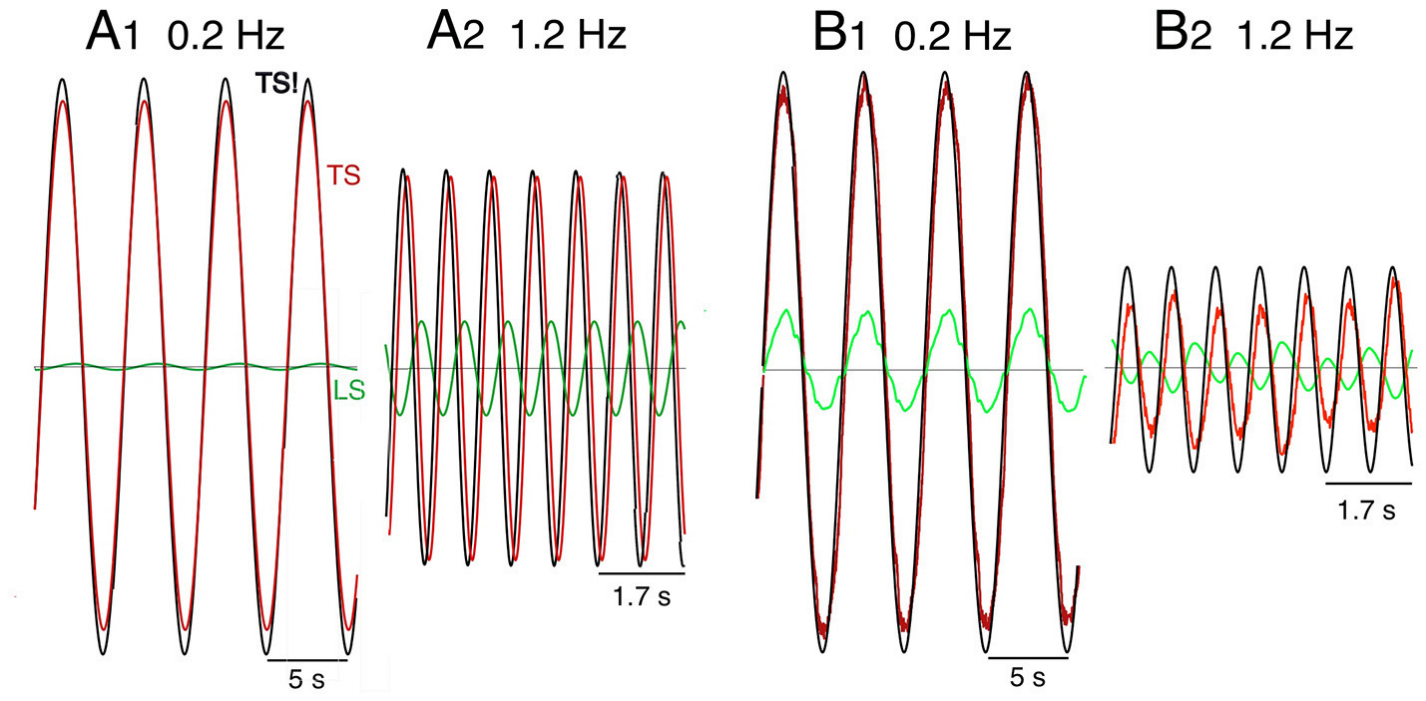
**Model simulation (A)** and robot experiments **(B)** of internally generated (“voluntary”) sinusoidal rotations of desired trunk-space angle (TS!). Dynamic effects on leg-in-space (LS) would ideally be absent (see text). Note that the support surface is here not rotating, so that desired ankle angle $q_{1}^{d}$ is equal to leg in space, LS, while $q_{2}^{d}=TS!-LS!$ (i.e., using a notation that is more intuitive in the following).

Proactive TS and LS movements were performed in addition to the robot experiments also in model simulations. A first overview was obtained with desired sinusoidal TS movements in space coordinates. Figure 7 shows the “voluntary” signals (desired trunk-space angle, TS!) in comparison with the executed movements signal (TS) for 0.2 and 1.2 Hz sine frequency (A, simulation data; B, robot data). At 0.2 Hz, only a very small coupling effect of TS on LS is visible in the simulations (A1), whereas a small in-phase reaction occurs in the robot (B1). At 1.2 Hz, the evoked LS excursions are increased and shifted toward counter-phase already in the simulation (A2) and more so in the robot (B2).

**Figure 8:**
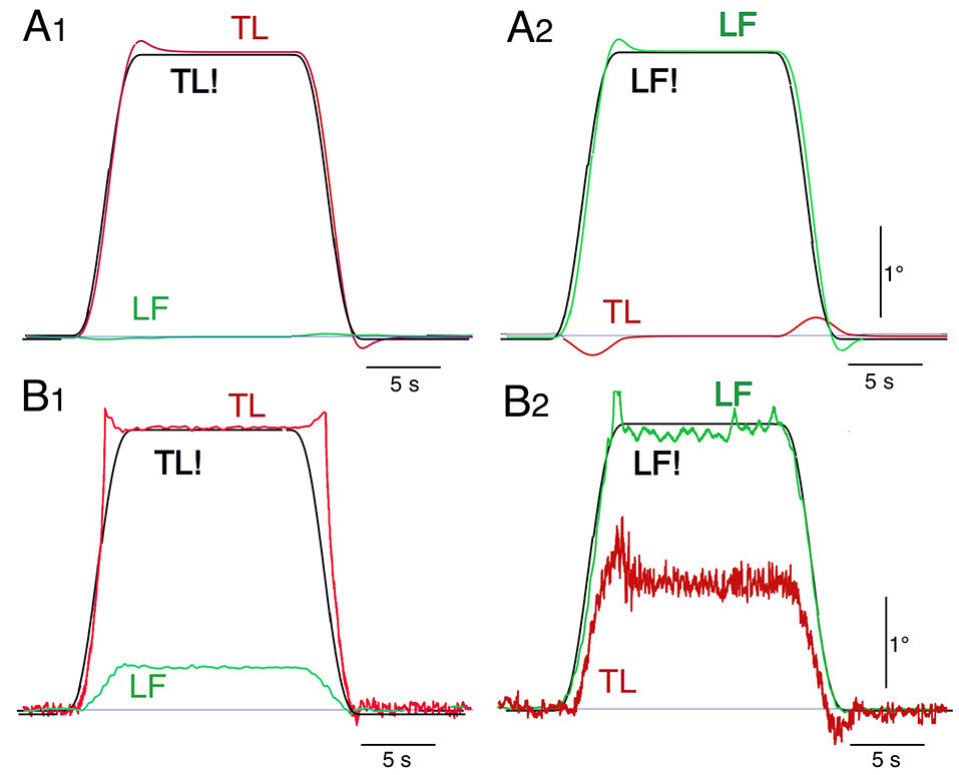
**Model simulation (A)** and robot experiments **(B)** of internally generated (“voluntary”) rotations with ‘raised cosine velocity profile’ (amplitude, 4°; dominant frequency, 0.2 Hz) of TL (desired, TL!) in **(A1,B1)** and of LF (desired, LF!) in **(A2,B2)**.

Noting that the separation between dynamic and static effects and the use of space coordinates Pcomplicate the interpretation of the robots behavior, movement commands with smoothed ramp-like waveform (raised cosine velocity function, see Appendix 1 in Supplementary Material) and proprioceptive coordinates were used in the simulations and robot experiments (Figure 8). Stable stance was obtained in both scenarios. In the model simulations a desired trunk-leg angle signal of 4° (TL! = 4°) leads to a slight TL overshoot and a weak dynamic LF counter excursion (A1). With a desired leg-foot angle (LF!) as command a slight LF overshoot and a clearly larger transient dynamic TL counter responses occurred (A2). In the corresponding robot experiments, the resulting TL movement also shows a dynamic response, mainly attributed to static friction effects (B1). LF showed no considerable dynamic effect, but a static excursion in TL lean direction. With a desired LF command, the resulting LF lean movement showed a dynamic overshoot (B2). The effect on TL consisted of very small dynamic counter-effects and a relatively large static excursion in the direction of LF of approximately 5.5°. The large TL response is mainly due to the relatively large weight of the trunk. It should be noticed that the used controller (PD) does not guarantee a null static error in the general case. This is more evident in robot experiments (B2) than in simulations (A2) where the control parameters can be perfectly tuned to the system. Taken together, dynamic coupling in simulations and the robot experiments were not completely abolished, but strongly reduced (e.g., A2), while gravitational torque effects where prominent, this mainly when the control operated in proprioceptive coordinates (Figure 8) and less so in space coordinates (Figure 7).

### Reactive responses to external disturbances

**Figure 9:**
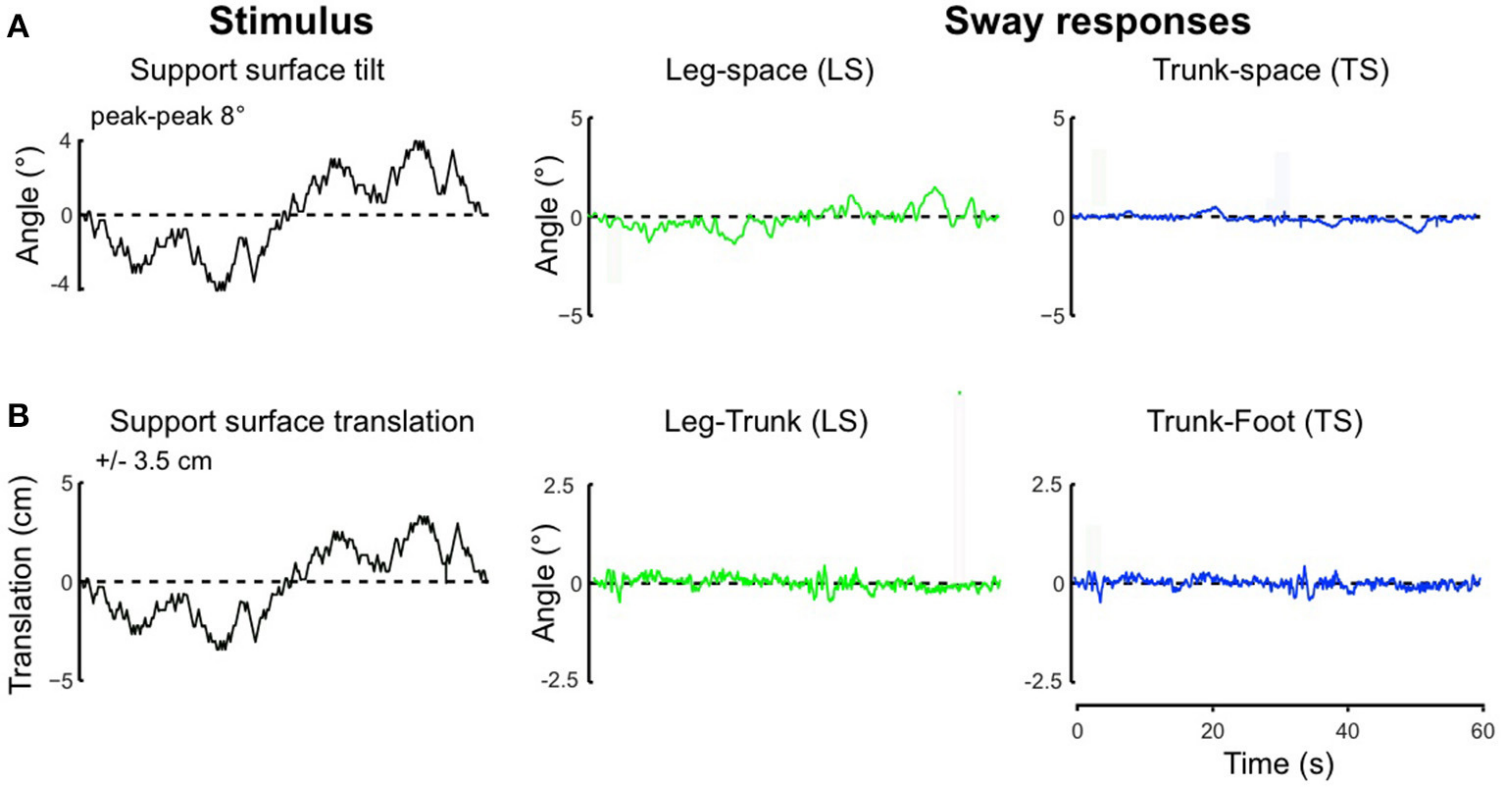
**Time series of stimulus and sway responses of the robot to support surface tilt** (**A**, with vestibular function; leg-in-space tilt, LS, and trunk-in-space tilt, TS, in space coordinates) and support surface translation (**B**, without vestibular function; leg-in-space tilt, LS, and trunk-in-space tilt, TS, in platform coordinates) using the PRTS stimulus waveform.

**Figure 10:**
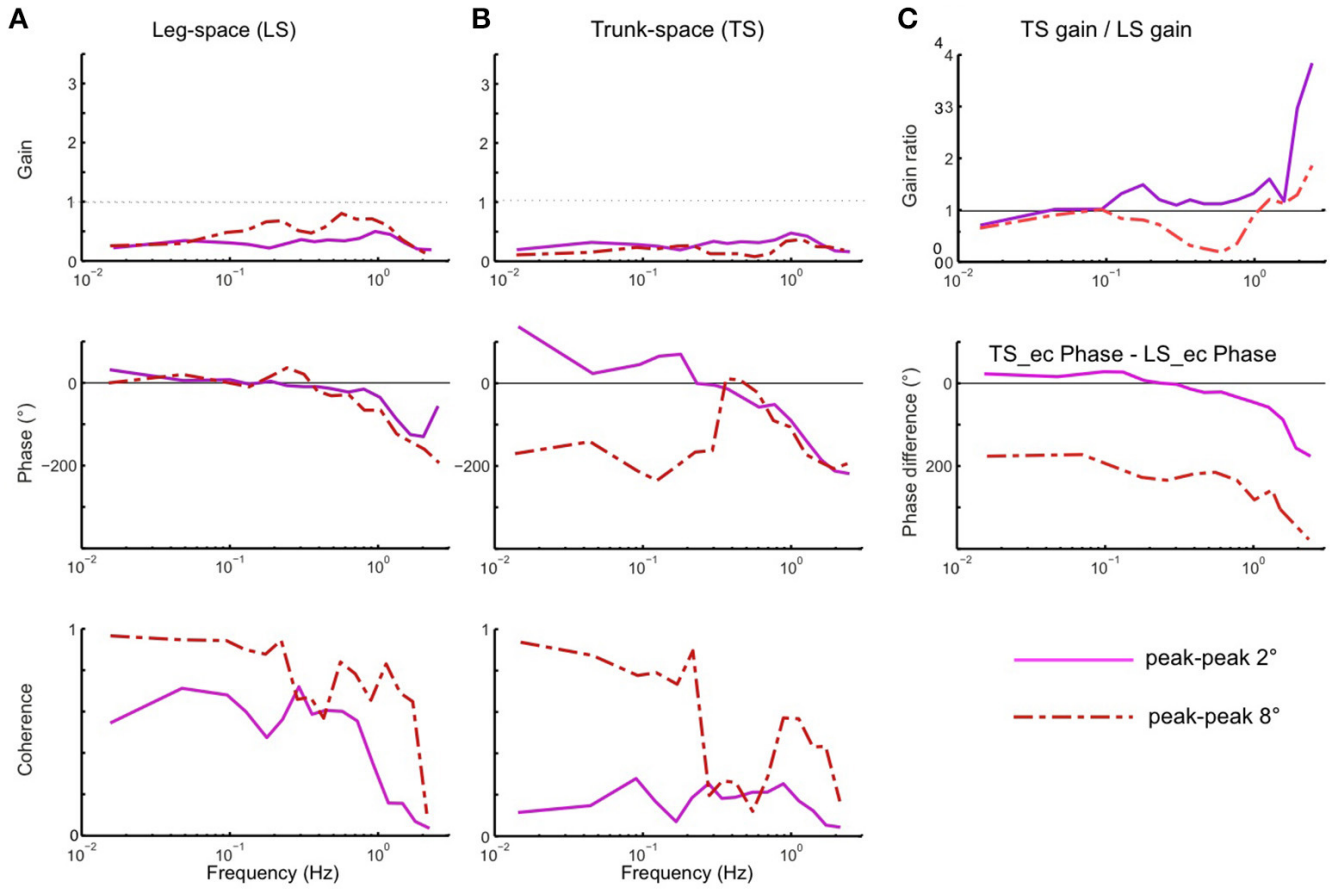
**(A–C)** Sway responses to support surface tilt of the robot with vestibular input presented in terms of frequency response functions (FRF) of LS to tilt **(A)**, TS to tilt **(B)**, and the ratio curves of TS gain to LS gain and difference curves between TS phase and LS phase **(C)** (PRTS stimulus of pp 2° and pp 8°).

**Figure 11:**
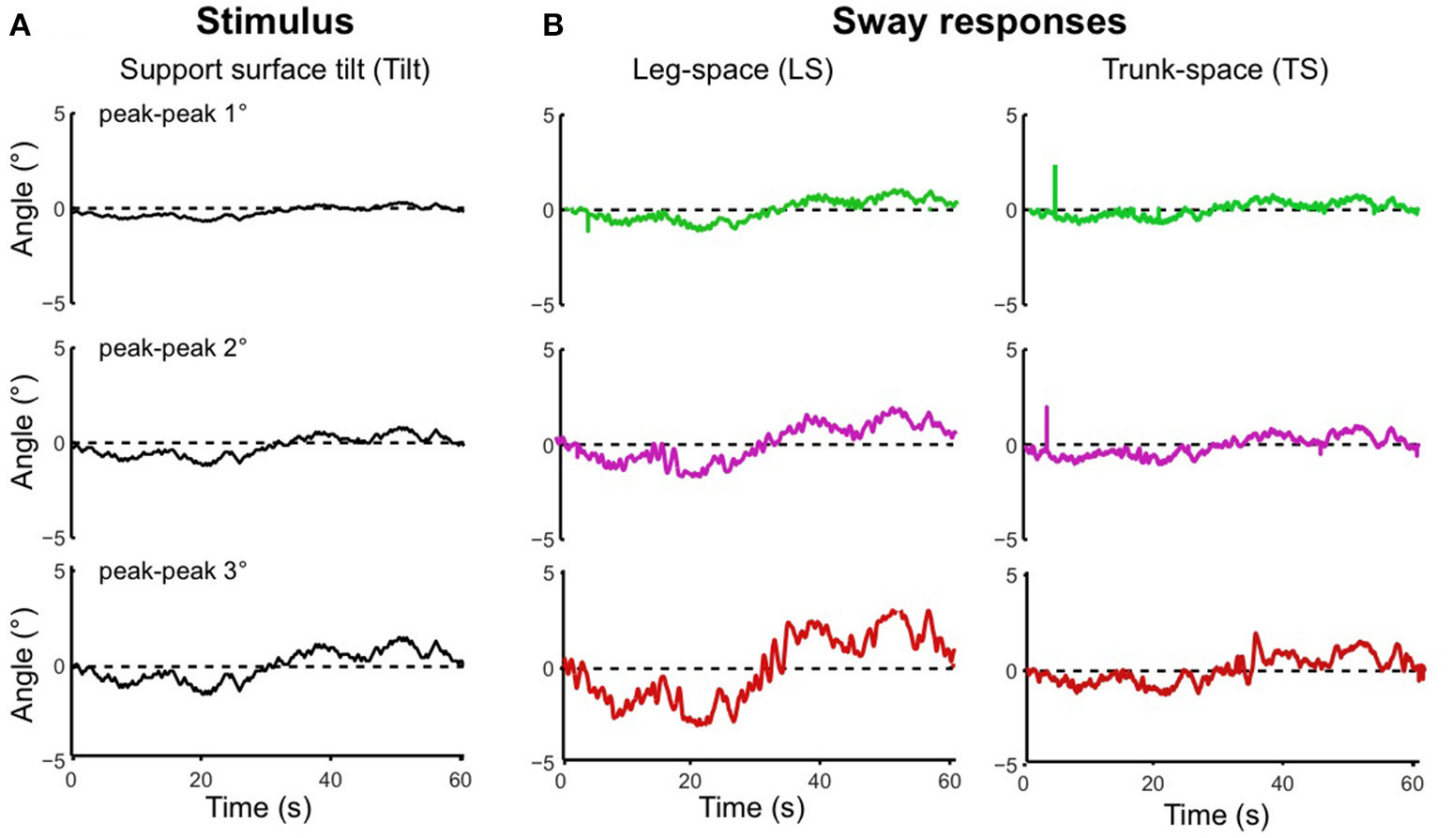
**Time series of stimulus and sway responses of the robot ***without*** vestibular function to support surface tilt. (A)** PRTS tilt stimuli of peak-peak amplitudes of 1°, 2°, and 3°. **(B)** Responses of leg in space, LS, and trunk in space, TS. Limiting tilt peak-peak amplitudes to 3° prevented falling.

**Figure 12:**
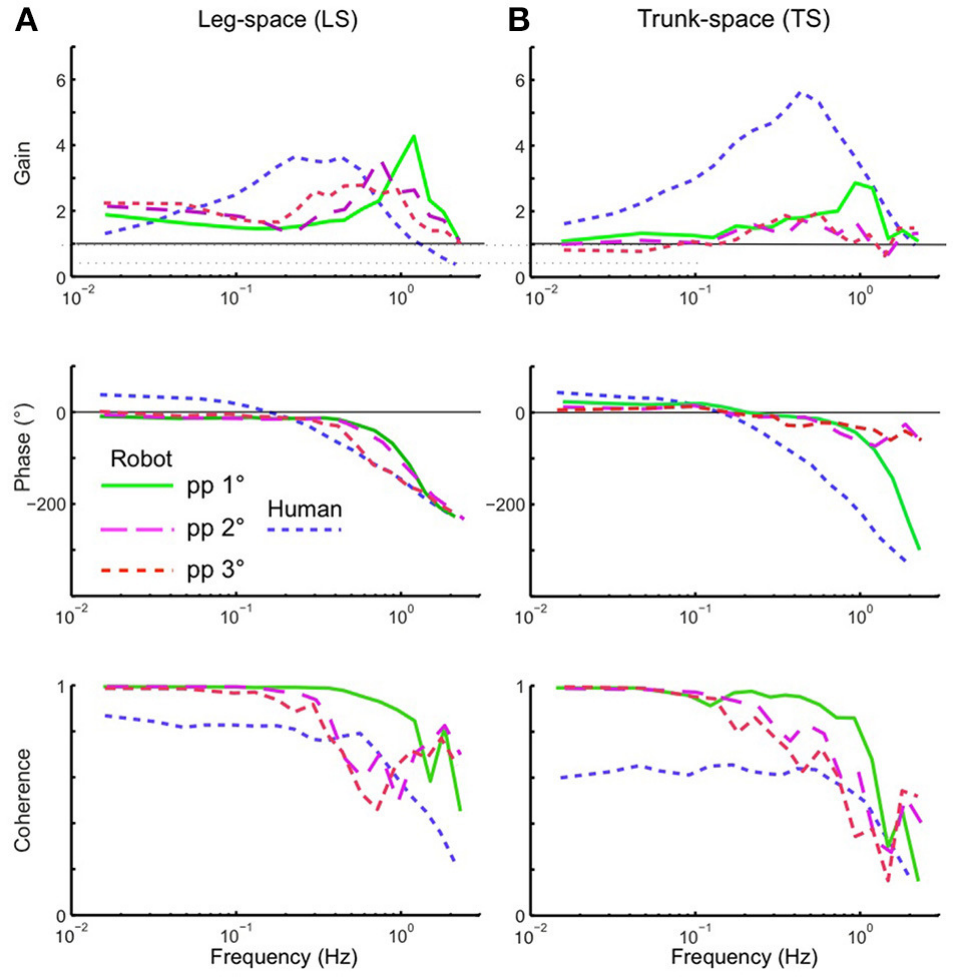
**Balancing of stance on tilting support surface of the robot ***without*** vestibular function (control is exclusively proprioceptive)**. Shown are frequency response functions (FRFs) and coherence functions for leg in space (LS; **A**) and trunk in space (TS; **B**). Superimposed are human data for comparison (mean of 5 vestibular-loss subjects, eyes closed).

Using the pseudo-random ternary sequence (PRTS) stimulus allows to analyze externally evoked LS and TS sway responses over a broad spectrum of frequencies (Hettich et al., 2014; adopted from Peterka, 2002). The method allows analyzing the data in the frequency domain in terms of frequency response functions (FRFs) and coherence functions (see Appendix 1 in Supplementary Material). Examples of the time series of the stimulus and responses in the sagittal plane are given in Figure 9A for support surface tilt with vestibular input (control of leg segment operated in coordinates of gravitational space) and in Figure 9B for support surface translation (control operated in joint coordinates). Note that the robot successfully maintains balance with relatively small angular leg and trunk excursions in the two experiments shown and in other balancing experiments performed (see also Film in Supplementary Materials). The FRF results for the tilt experiments with pp 2° and pp 8° are given in Figure 10. They show that the robot keeps the orientation of the legs in space and the trunk in space upright. The robot was able to maintain balance also without vestibular input (control of leg segment operated with respect to the feet) when the support surface tilt amplitude was reduced to 3° or smaller (Figures 11, 12), a performance that qualitatively is similar to that of vestibular loss human subjects (see Discussion).

## Discussion

Theoretically, the EM concept is a relatively simple and efficient method to cope in a multi-DoF system with the coupling forces between mechanically linked segments. This study tested whether the EM concept is able to cope with coupling forces also in the control of a real-world technical device such as a humanoid robot, in which the control faces non-ideal properties such as noisy, inaccurate and non-linear sensors, friction and backlash, etc. These real world conditions may challenge control robustness in face of human-like feedback time delays. The robot experiments demonstrate that the EM control method copes considerably well with the real-world properties in a humanoid robot with human-like anthropometrics and equipped with human-inspired sensors and actuators. Therefore, we consider the EM control method a valid candidate that should be considered when making inferences on which method humans may use for their sensorimotor control. In the following, we first briefly address general issues of the EM concept before considering our experimental findings and consider alternatives to the EM control method.

Considering the EM method in this study was not meant to reduce the number of degrees of freedom of the system or to solve a redundancy problem, because the number of DOFs is the same when controlling joints or EM spaces. Rather, the benefit of using the EM concept is a control simplification in that multiple EMs can simultaneously be controlled dynamically independently of each other. For example, with a trunk bending forward the robot may simultaneously move the leg segment backwards in order to balance the COM over its feet as base of support (corresponding to a kinematic synergy), but alternatively may maintain the leg segment vertical (coping with forward shift of the COM through corresponding ankle torque). Generally, it should be noted that EMs are not synergies observable in the space of joint angles, but a step in the design of a control system that can produce arbitrary poses and trajectories (within the limit of the robot's dynamic responses). Independent PD-control of separate EMs allows clearly longer time delays in the feedback loop than the independent PD control of separate joints. For example, the Lyapunov index showed that the independent PD-controls of separate EMs in PostuRob II is stable up to a time delay of Δ*t* = 200 ms, while the limit is Δ*t* = 150 ms using independent controls of separate joints (see Appendix 5 in Supplementary Material). Conceivably, in a more complex control system such as the human one, additional or other mechanisms may contribute to control stability in face of long time delays.

The definition of the EMs implies a linearization of the system. With the control of upright body posture, a natural choice for the linearization point is the vertical position. Principally, however, the system has previously been shown to work also for a wider range of movements, exploiting successive linearization points as described for arm movements (Frolov et al., 2013). It remains to be shown how complexity increases when the EM method has to deal continuously with large operative spaces.

Despite the fact that the design of Posturob II takes into account the human anthropometry, time delays in the control loop and some human-inspired sensors and actuators it ignores several known constituents of the human posture control system such as load-related proprioceptive sensors (Dietz, 1998), foot deformation (Wright et al., 2012), some minor role of the knee joints (Alexandrov et al., 2001b), and more. Even though ignoring these parts of the human posture control, the EM approach provided stable maintenance of posture and movements in the humanoid robot. Similarly, previous feedback control models, using in the absence of visual information only joint angle proprioceptive and vestibular sensory inputs, were sufficient to quantitatively describe human responses to moderate support surface tilt stimuli in the sagittal plane, as also shown with other control models such as the independent channel (IC) model (SIP biomechanics Peterka, 2002) or the disturbance estimation and compensation (DEC) model (DIP biomechanics; (Hettich et al., 2014); this study includes a direct comparison between data of humans and of Posturob II using the human-derived model). The model of Park et al. (2004); see also (Kuo and Zajac, 1993), one of several currently available models of posture control, used proprioceptive linear full state feedback control to describe human responses to backwards translation and found only moderate improvement when increasing complexity from a 2-segment to a 4-segment model.

The main result of the present study is the experimental demonstration that the EM method copes well with a PD-control of a “real-world” mechanical anthropomorphic robot. The feedback loop parameters for the independent control in each EM were calculated from the robot anthropometrics, including human-like feedback time delays. Other characteristics of the robot as a “real-world” system, which typically are not exactly known such as friction, nonlinearities, noise, backlashes, inaccuracies, etc., were ignored. These unaccounted factors led to clear deviations of measured results from model predicted results, but influenced relatively little the overall characteristics of the movements and did not contradict the hypothesis that the EM concept can provide in principle stable performance of the robot. This applies to both proactive movements and reactive balancing of stance during unforeseen external disturbances in the sagittal plane (such as horizontal translation of the support surface and, when vestibular information was included into the control, support surface tilt).

Another aspect to be considered in the present approach was that the EM implementation aimed at an optimal stability of the control (see Section Theoretical Analysis of Control Stability). This does not imply that thereby the robot's postural responses automatically become similar to human subjects. In other words, optimizing the EM control for Posturob II does not mean that the robot's postural responses become human-like because of its human-inspired sensors and actuators alone. The present approach differs from that in a previous study, which also used Posturob II (Hettich et al., 2014). There, human-like postural responses of the robot were obtained by fitting the control parameters of a posture control model, which later controlled the robot, to the human responses. Still, we considered it as interesting to compare in the present context the robot data with human data to visualize differences in the sway response behavior. To this end, we superimposed on the robot's frequency response functions shown in Figure 12 the results obtained with the same stimulus and set up obtained from a group of vestibular loss human subjects with eyes closed. The reason for this choice was to consider an especially simple control that uses mainly proprioceptive sensors (in humans possibly including force cues; Mergner et al., 2009). Outstanding differences between human and robot data are larger human sway responses in the mid-frequency range of the PRTS stimulus, a slightly different phase behavior, and smaller coherence in the low to mid frequency range, possibly indicating higher sensory or motor noise (or more general, effects not fully taken into account by the model). Future studies may use parameter identification methods in order to fit the EM concept to human data.

### Possible implications for robotics

*Kinematic* synergies are widely used in humanoid robotics, typically with the purpose to simplify movement control. *Kinetic* synergies, i.e., predefined coordination between joint torques, although considered in numerous human studies (Prattichizzo et al., 2010; Shim et al., 2010), are rather sparsely implemented in explicit form in robots. As to the EM, they are formally kinematic synergies, yet the variables η are kinetic synergies defined by the coefficients of the matrix ***U***. Formally, EM are defined to be coupling-free and to cover the whole space of joint motion. They are not producing a simplification of the control in terms of DOFs, yet, being dynamically independent, they simplify the structure of the control problem to the case of the control of several independent SISO systems. This can be advantageous for robotic control both in simplifying the design of the control system and to improve robustness with respect to modifications in control parameters, since modifications to one of the SISO controllers does not affect the others. This can be important in cases when the control parameters need to be dynamically adjusted, e.g., to improve balance performance during different tasks or in different scenarios.

However, the simplification of the control design by using SISO controllers comes at the cost of designing the transformation matrices ***U*** and ***W***. This transformation requires a reliable model of the body dynamics, which may be problematic with certain tasks such as with full body control of a humanoid that involves a large number of DOFs. A further limitation is given by the system linearization required to define the Eigen movements, since this implies the necessity to linearize the system in different points of the control space when trying to cover a wide range of motions and/or when involving several distributed DOFs in complex behaviors such as walking. Overall, considering advantages versus limitations of the EM concept applied to robotics, we conceive that whole body control tasks like balancing upright stance would profit from it, because the limited range of motion makes errors due to linearization negligible. We would expect profits also for applications in which the dynamic requirements of the task and the presence of time delays make the coupling forces highly relevant, such as in the control of fast arm reaching (Frolov et al., 2013). In case that the number of DOFs involved in a task is large so as to make full state solutions non trivial, use of the EM approach with accurate definition of ***W*** and ***U*** still may be practicable, this especially if the control task is reduced to a subset of variables by means of integration of task specific constraints.

## Conclusions

A major conclusion from the present experiments refers to the robot experiments as an experimental tool when studying the human sensorimotor control system. Using a humanoid robot for comparing different bio-inspired control concepts with each other on the same robot will help to define criteria for presumed human-likeness of control algorithms—with potential benefits also for use in humanoid robotics and user acceptance in robotic neurorehabilitation. In doing so, the robot experiments provide a valuable “real world” test that complements model simulations, especially in addressing the problem of control stability in face of human time delays. Finally, an experience from this study is that “learning by doing” in the robot experiments provides inspirations also for the research of the human control system.

## Ethics statement

All subjects gave their informed written consent to the study that was approved by the Ethics Committee of the Freiburg University Clinics, in accordance with the Declaration of Helsinki.

## Author contributions

All authors performed the experiments and collected the data. GH, VL, and TM performed the computer simulations and analyzed the robot and human data. All authors contributed to the interpretation of the data and contributed to writing the manuscript. All authors approved the final version of the manuscript for submission.

## Funding

The work was supported by the Russian Science Foundation, grant 16-15-00219, for the author AF and by the European Commission FP7 Grant 600698 H2R and 610454 EMBalance for the authors GH, TM, and VL.

### Conflict of interest statement

The authors declare that the research was conducted in the absence of any commercial or financial relationships that could be construed as a potential conflict of interest.

## References

[B1] Alexandrov A. V. Frolov A. A. (2011). Closed-loop and open-loop control of posture and movement during human trunk bending. Biol. Cybern. 104, 425–438. 10.1007/s00422-011-0442-x 21710218

[B2] Alexandrov A. V. Frolov A. A. Horak F. B. Carlson-Kuhta P. Park S. (2005). Feedback equilibrium control during human standing. Biol. Cybern. 93, 309–322. 10.1007/s00422-005-0004-1 16228222 PMC1430400

[B3] Alexandrov A. V. Frolov A. A. Massion J. (2001a). Biomechanical analysis of movement strategies in human forward trunk bending. I. Modeling. Biol. Cybern. 84, 425–434. 10.1007/PL00007986 11417054

[B4] Alexandrov A. V. Frolov A. A. Massion J. (2001b). Biomechanical analysis of movement strategies in human forward trunk bending. II. Experimental study. Biol. Cybern. 84, 435–443. 10.1007/PL00007987 11417055

[B5] Barin K. (1989). Evaluation of a generalized model of human postural dynamics and control in the sagittal plane. Biol. Cybern. 61, 37–50. 10.1007/BF00204758 2742913

[B6] Bastian A. J. (1997). Mechanisms of ataxia. Phys. Ther. 77, 672–675. 10.1093/ptj/77.6.672 9184691

[B7] d'Avella A. Giese M. Ivanenko Y. P. Schack T. Flash T. (2015). Editorial: modularity in motor control: from muscle synergies to cognitive action representation. Front. Comput. Neurosci. 9:126. 10.3389/fncom.2015.00126 26500533 PMC4598477

[B8] Dietz V. (1998). Evidence for a load receptor contribution to the control of posture and locomotion. Neurosci. Biobehav. Rev. 22, 495–499. 10.1016/S0149-7634(97)00035-3 9595560

[B9] Flash T. Bizzi E. (2016). Cortical circuits and modules in movement generation: experiments and theories. Curr. Opin. Neurobiol. 41, 174–178. 10.1016/j.conb.2016.09.013 27736649

[B10] Frolov A. A. Biryukova E. V. Bobrov P. D. Mokienko O. A. Platonov A. K. Pryanichnikov V. E. (2013). Principles of neurorehabilitation based on the brain-computer interface and biologically adequate control of the exoskeleton. Hum. Physiol. 39, 196–208. 10.1134/S0362119713020035 23789390

[B11] Frolov A. A. Dufossé M. Øízek S. Kaladjian A. (2000). On the possibility of linear modelling the human arm neuromuscular apparatus. Biol. Cybern. 82, 499–515. 10.1007/s004220050603 10879434

[B12] Frolov A. A. Prokopenko R. A. Dufosse M. Ouezdou F. B. (2006). Adjustment of the human arm viscoelastic properties to the direction of reaching. Biol. Cybern. 94, 97–109. 10.1007/s00422-005-0018-8 16344944

[B13] Hettich G. Asslander L. Gollhofer A. Mergner T. (2014). Human hip-ankle coordination emerging from multisensory feedback control. Hum. Mov. Sci. 37, 123–146. 10.1016/j.humov.2014.07.004 25150802

[B14] Kuo A. D. (1995). An optimal control model for analyzing human postural balance. IEEE Trans. Biomed. Eng. 42, 87–101. 10.1109/10.362914 7851935

[B15] Kuo A. D. Zajac F. E. (1993). Human standing posture: multi-joint movement strategies based on biomechanical constraints. Prog. Brain Res. 97, 349–358. 10.1016/S0079-6123(08)62294-3 8234760

[B16] Kurtzer I. Pruszynski J. A. Scott S. H. (2009). Long-latency responses during reaching account for the mechanical interaction between the shoulder and elbow joints. J. Neurophysiol. 102, 3004–3015. 10.1152/jn.00453.2009 19710379

[B17] Kurtzer I. Trautman P. Rasquinha R. J. Bhanpuri N. H. Scott S. H. Bastian A. J. (2013). Cerebellar damage diminishes long-latency responses to multijoint perturbations. J. Neurophysiol. 109, 2228–2241. 10.1152/jn.00145.2012 23390311 PMC3628027

[B18] Lacquaniti F. Soechting J. F. (1986). EMG responses to load perturbations of the upper limb: effect of dynamic coupling between shoulder and elbow motion. Exp. Brain Res. 61, 482–496. 10.1007/BF00237573 3956610

[B19] Massion J. (1992). Movement, posture and equilibrium: interaction and coordination. Prog. Neurobiol. 38, 35–56. 10.1016/0301-0082(92)90034-C 1736324

[B20] Mergner T. (2012). Postural control by disturbance estimation and compensation through long-loop responses, in Routledge Handbook of Motor Control and Motor Learning, eds Gollhofer A. Taube W. Nielsen J. B. (London: Routledge), 50–70.

[B21] Mergner T. Maurer C. Peterka R. J. (2003). A multisensory posture control model of human upright stance. Prog. Brain Res. 142, 189–201. 10.1016/S0079-6123(03)42014-1 12693262

[B22] Mergner T. Schweigart G. Fennell L. (2009). Vestibular humanoid postural control. J. Physiol. Paris 103, 178–194. 10.1016/j.jphysparis.2009.08.002 19665555

[B23] Ott C. Dietrich A. Roa M. A. (2014). Torque-based multi-task and balancing control for humanoid robots, in International Conference on Ubiquitous Robots and Ambient Intelligence (URAI) (Kuala Lumpur), 143–144.

[B24] Ott C. Henze B. Hettich G. Seyde T. N. Roa M. A. Lippi V. (2016). Good posture, good balance: comparison of bioinspired and model-based approaches for posture control of humanoid robots. IEEE Robot. Autom. Magazine 23, 22–33. 10.1109/MRA.2015.2507098

[B25] Park S. Horak F. B. Kuo A. D. (2004). Postural feedback responses scale with biomechanical constraints in human standing. Exp. Brain Res. 154, 417–427. 10.1007/s00221-003-1674-3 14618285

[B26] Peterka R. J. (2002). Sensorimotor integration in human postural control. J. Neurophysiol. 88, 1097–1118. 10.1152/jn.00605.2001 12205132

[B27] Prattichizzo D. Malvezzi M. Bicchi A. (2010). On motion and force controllability of grasping hands with postural synergies, in Proceedings of Robotics: Science and Systems (Zaragoza)

[B28] Shim J. K. Hooke A. W. Kim Y. S. Park J. Karol S. Kim Y. H. (2010). Handwriting: hand-pen contact force synergies in circle drawing tasks. J. Biomech. 43, 2249–2253. 10.1016/j.jbiomech.2010.04.033 20488445

[B29] Welch T. D. Ting L. H. (2008). A feedback model reproduces muscle activity during human postural responses to support-surface translations. J. Neurophysiol. 99, 1032–1038. 10.1152/jn.01110.2007 18094102

[B30] Wright W. G. Ivanenko Y. P. Gurfinkel V. S. (2012). Foot anatomy specialization for postural sensation and control. J. Neurophysiol. 107, 1513–1521. 10.1152/jn.00256.2011 22157121 PMC3311689

