# Supplementary material for: Human-Inspired Eigenmovement Concept Provides Coupling-Free Sensorimotor Control in Humanoid Robot
Source: Front Neurorobot. 2017 Apr 25;11:22. doi: 10.3389/fnbot.2017.00022 (PMC5403929; doi:10.3389/fnbot.2017.00022)
Supplement: Supplementary file 2 [file Appendix1.docx]

# Appendix 1

*Robot parameters*. The lower link (legs) length and mass L_1_ = 0.86 m, m_1_ = 14 kg, the estimated distance of the center of mass from its distal end c_1_ = 0.43 m, the estimated moment of inertia about the center of mass J_1_ = 3.45 kg m^2^. The upper link (trunk) respectively – L_2_ = 0.58 m, m_2_ = 37 kg, c_2_ = 0.35 m and J_2_ = 4.15 kg m^2^. The coefficients of the inertial and gravity matrices **B**_0_ and **G**_0_ in equation (2) can be calculated by formulas presented, for example, in (Alexandrov et al. 2001a): $B_{011}=64.36$N m s^2^rad^-1^, $B_{012}=B_{021}=$N m s^2^rad^-1^, $B_{022}=8.68$N m s^2^rad^-1^, $G_{011}=498.25$ N m rad^-1^, $G_{012}=G_{021}=G_{022}=127.09$N m rad^-1^.

*Methods of robot experiments.* The methods applied previously in human and robot experiments were used (Hettich et al. 2014). In short, the robot stood in standard parallel stance on a custom-made motion platform in a posture control laboratory that was (a) tilted in the sagittal plane such that the rotation axis of the platform passed through the ankle joint axes, and (b) horizontally translated in the sagittal plane. Leg/trunk sways around the ankle/hip joints and platform motions were measured using an opto-electronic device with active markers (Optotrak 3020; Waterloo, Canada). The data were recorded using an A/D converter with 100 Hz sampling frequency and custom-made software programmed in LabView (National Instruments, Austin, USA). Recorded data were exported to Matlab (The MathWorks, Natick, USA) for further analysis. Angular excursions of leg-space angle, *LS*, and trunk-space angle, *TS*, were calculated from measured heights of the hip and shoulder markers using trigonometric functions.

Tilt evoked *LS* and *TS* sway responses to support surface tilt were further analyzed in the frequency domain in terms of frequency response functions (FRFs) and coherence functions (compare Peterka, 2002). The discrete Fourier transforms of the tilt stimulus and the measured *LS* and *TS* and sway responses were calculated using the *dft* function implemented in Matlab. The spectra were calculated at 16 frequencies, approximately equally spaced on a logarithmic scale from 0.017 to 2.2 Hz (Peterka, 2002) and averaged across the 6 PRTS cycles. FRFs were calculated by dividing the cross power spectra of stimulus and response by the power spectra of the stimulus. Tilt-evoked FRFs were expressed as gain (absolute value of the FRF) and phase (inverse tangent of the FRF). Gain is given by the amplitude ratio between sway response and tilt stimulus. A gain above unity indicates that the sway response amplitude exceeds the stimulus amplitude at the given frequency and a gain below unity that the sway response amplitude is smaller than the stimulus amplitude. The phase gives the temporal relation between stimulus and response. Coherence functions were calculated by dividing the squared absolute values of the cross power spectrum by the product of the stimulus and response power spectra. Coherence function values are a measure of the frequency dependent signal to noise ratio of the system. 95% confidence limits of gain and phase curves were calculated using bootstrapping methods with 1000 bootstrap samples at each smoothed frequency point (Zoubir and Boashash, 1998). Additionally, the ratio of the *LS* to *TS* gain values and the difference between *LS* and *TS* phase values were calculated (Kiemel, Elahi, & Jeka, 2008). Finally, for a first survey, we applied stimuli with sine waveform and ‘raised cosine velocity’ waveform (bell-shaped velocity profile, *v*(t) = –*A* · *f* · cos(2π*ft*) + *A* · *f*, where *t* is time, *A* is angular displacement and *f* is dominant frequency).

For comparison with the robot data, five young subjects (two females; mean 18.8 years ± 0.8 SD) with chronic bilateral loss of vestibular functions participated in the study. Vestibular loss was diagnosed by clinical examinations (e.g. balance problems when standing on foam rubber with eyes closed), case histories (e.g. meningitis and ototoxic medication in childhood) and electronystagmography (absence of caloric and rotation-evoked nystagmus). Apart from hearing problems, subjects were healthy and showed no neurological symptoms. All subjects gave their informed written consent to the study that was approved by the Ethics Committee of the Freiburg University Clinics, in accordance with the Declaration of Helsinki.

# Appendix 2

*Analysis of control stability.* The optimal PD-controller parameters were obtained by calculating the ranges in the space of *S*^eig^ and *V*^eig^ in which the Lyapunov index does not exceed the given values *α*. For each given *α*, the range is defined by the crosscut of two branches of solution of (11). One branch corresponds to the real roots of (11), i.e. to the condition *μ* = *α*. If this condition is satisfied then *S*^eig^ and *V*^eig^ satisfy equation

| $S^{eig}+\alpha V^{eig}{+1+(\lambda\alpha}^{2}-1)e^{\alpha\Delta t}=0$ | (A2.1) |
| --- | --- |

which describes the straight-line in the (*V*^eig^ × *S*^eig^)-plane. The second branch corresponds to the roots *μ* with non-zero imaginary parts *ω*. Then the values *S*^eig^ and *V*^eig^ for a given root *μ = α + iω* can be found as the solution of the system of two real equations

|  |  |
| --- | --- |

$S^{eig}\cos\left( \omega\Delta t \right)+V^{eig}\left[ \alpha\cos\left( \omega\Delta t \right)+\omega\sin\left( \omega\Delta t \right) \right]=\left[ 1+\lambda\left( \omega^{2}-\alpha^{2} \right) \right]e^{\alpha\Delta t}-\cos\left( \omega\Delta t \right)$ $(A2.2)$

| $S^{eig}\frac{sin\left( \omega\Delta t \right)}{\omega}+V^{eig}\left[ \alpha\frac{sin\left( \omega\Delta t \right)}{\omega}-cos\left( \omega\Delta t \right) \right]= 2\lambda\alpha e^{\alpha\Delta t}-\frac{sin\left( \omega\Delta t \right)}{\omega}$ | (A2.3) |
| --- | --- |

The plane region (*V*^eig^ × *S*^eig^) in which the Lyapunov indices are less than *α* will henceforth be called *α*-range. Within the *α*-range for *α* = 0 the real parts of all the roots of (11) are negative and the system is stable. Outside this range, the system is unstable. The region of stability is restricted from above and from below for both parameters *S*^eig^ and *V*^eig^ and this region decreases with the increase of time delay Δ*t*. For a given value of *λ* there exists a maximum value ∆*t*_max_ when the region of stability disappears, i.e. the stable movement is impossible under the delay Δ*t* > ∆*t*_max_. For example ∆*t*_max_ = 0.44 s when *λ* = 0.1 s^2^ and ∆*t*_max_ = 0.20 s when *λ* = 0.02 s^2^. Similarly, for given values of *λ* and Δ*t*, there exists a minimum value *α*_min_ where the *α*-range disappears. This point defines the optimal parameters $S_{opt}^{eig}$ and $V_{opt}^{eig}$ of the PD-controller feedback loop that provide the fastest possible response of the system to external perturbations and the minimum delay in the transformation of the desired kinematics to the actual one.

# Appendix 3

Let transfer function $\mathbf{F}^{\mathrm{XY}}$ be the function that describes the transformation of two-dimensional signal $\mathbf{X}=\mathbf{X}_{0}\exp\left( i\omega t \right)$ to two-dimensional signal $\mathbf{Y}=\mathbf{Y}_{0}\exp\left( i\omega t \right)$. Vectors $\mathbf{X}_{0}$ and $\mathbf{Y}_{0}$ are calculated as $\mathbf{X}_{0}=\left\langle\boldsymbol{X\cdot}\exp\left( -i\omega t \right) \right\rangle$ and $\mathbf{Y}_{0}=\left\langle\boldsymbol{Y\cdot}\exp\left( -i\omega t \right) \right\rangle$ where the brackets $\left\langle\cdots\right\rangle$ mean the time averaging. Vectors $\mathbf{X}_{0}$ and $\mathbf{Y}_{0}$ obtained for each ratio of the desired joint angle amplitudes and a given frequency create the matrices $\mathbf{M}_{X}$ and $\mathbf{M}_{Y}$ as their columns. Transition function $\mathbf{F}^{\mathrm{XY}}$ can be then calculated as $\mathbf{F}^{\mathrm{XY}}=\mathbf{M}_{Y}\mathbf{M}_{X}^{+}$where $\mathbf{M}_{X}^{+}\mathbf{=}\mathbf{M}_{X}^{T}\left( \mathbf{M}_{X}\mathbf{M}_{X}^{T} \right)^{-1}$ is the pseudoinvers of $\mathbf{M}_{X}$**.** Due to the difference of amplitude ratios of the desired joint angles in five experimental recordings for a given frequency, two-by-two matrix $\mathbf{M}_{X}\mathbf{M}_{X}^{T}$ is not singular. In principle, only two experiments with different ratios of amplitudes for the signal $\mathbf{X}(t)$ are enough to estimate nonsingular matrix $\mathbf{M}_{X}\mathbf{M}_{X}^{T}$ (Boonstra et al., 2013). However because of the high level of noise in a real mechanical construction of Posturob II, we used five such ratios. Each complex coefficient

| $F_{ij}^{\mathrm{XY}}=\mathrm{Amp}\left( F_{ij}^{\mathrm{XY}} \right)\cdot\exp\left( i\cdot\mathrm{Phase}\left( F_{ij}^{\mathrm{XY}} \right) \right)$ | (A3.1) |
| --- | --- |

identifies the influence of the *j*-th component of signal $\mathbf{X}(t)$ to the *i*-th component of signal $\mathbf{Y}(t)$, where $\mathrm{Amp}\left( F_{ij}^{\mathrm{XY}} \right)$ and $\mathrm{Phase}\left( F_{ij}^{\mathrm{XY}} \right)$  are the amplitude and the phase transfer functions, respectively.

# Appendix 4

The Fourier transform of the dynamic equation (1) with regard to the expression for the control joint torques (6) has the form:

| $\mathbf{M}_{1}\mathbf{Q=}\mathbf{M}_{2}\mathbf{Q}^{d}$ | (A4.1) |
| --- | --- |

where $\mathbf{Q}$ and $\mathbf{Q}^{d}$ are the two-dimensional vectors of the Fourier transform for the actual and desired joint angles $\boldsymbol{q}(t)$ and $\boldsymbol{q}^{d}(t),$

| $\mathbf{M}_{1}=-\omega^{2}\mathbf{B}_{0}-\mathbf{G}_{0}\mathbf{+}\mathbf{M}_{t}\left( \mathbf{G}_{0}+\mathbf{S+}i\omega\mathbf{V} \right)$  $\mathbf{M}_{2}=\mathbf{M}_{t}\left( \mathbf{S+}i\omega\mathbf{V} \right)$ | (A4.2) |
| --- | --- |

**S** and **V** are stiffness and viscosity matrices in equation (2) expressed in terms of joint angles,

| $\mathbf{M}_{t}\boldsymbol{=}\left( \begin{matrix} \exp\left( -i\omega{\Delta t}_{A} \right) & 0 \\ 0 & \exp\left( -i\omega{\Delta t}_{H} \right) \end{matrix} \right)$ | (A4.3) |
| --- | --- |

and Δ*t*_А_, Δ*t* _H_ are the total delays in the transformation of the required control torques to the actual torques respectively in the ankle and hip joints. The transfer function for the desired joint angles to the actual joint angles is:

| $\mathbf{F}^{qq}=\mathbf{M}_{1}^{-1}\mathbf{M}_{2}$ | (A4.4) |
| --- | --- |

The hardware delays ${\Delta t}_{1}$ and ${\Delta t}_{2}$ in Posturob II were intentionally aligned by controller delays ${\Delta t}_{1}^{c}$ and ${\Delta t}_{2}^{c}$ so that ${\Delta t}_{A}={\Delta t}_{H}=\Delta t=0.1$s, which implies that $\mathbf{M}_{t}\boldsymbol{=}e^{-i\omega\Delta t}\mathbf{E}$, where **E** is two-by-two identity matrix.

# Appendix 5

Let the humanoid movement be performed under the PD-controller according to equation (6) in which matrices $\mathbf{S}$ and $\mathbf{V}$ are diagonal (that is torques in each joint are controlled independently) with diagonal elements $S_{1},S_{2},V_{1},V_{2}$. Then the secular equation has the form

| $\det\left( \mathbf{B}_{0}\mu^{2}-\mathbf{G}_{0}\mathbf{+}\left( \mathbf{G}_{0}+\mathbf{S} \right)e^{-\mu\Delta t}+\mathbf{V}\mu e^{-\mu\Delta t} \right)=0.$ | (A5.1) |
| --- | --- |

The roots of the secular equation were found numerically for given values ${\Delta t, S}_{1},S_{2},V_{1},V_{2}$ with PostuRob II mechanical parameters obtained experimentally. The Lyapunov index$\alpha\left( \Delta t, S_{1},S_{2},V_{1},V_{2} \right)$ was calculated as the maximum real part of the roots. The optimal Lyapunov index $\alpha_{\min}\left( \Delta t \right)$ was calculated for each given delay $\Delta t$ as minimum $\alpha$ over all possible values $S_{1},S_{2},V_{1},V_{2}$. The dependence $\alpha_{\min}\left( \Delta t \right)$ was compared with the optimal Lyapunov indexes obtained in the framework of eigenmovement approach. The latter approach yields much larger absolute values of Lyapunov index thus providing much less delays in transformation of the desired kinematics to the actual one. Moreover, under the independent PD-control of separate joints, the stable PD-control becomes impossible at $\Delta t>0.15 s$ while the EM approach yields the stable control for time delays up to $\Delta t=0.20 s$.

References

Boonstra, T.A., Schouten, A.C., and Van der Kooij, H. (2013). Identification of the contribution of the ankle and hip joints to multi-segmental balance control. *J. Neuroeng. Rehabil.* 10, 23*.*

Kiemel, T., Elahi, A.J. and Jeka, J.J., (2008). Identification of the plant for upright stance in humans: multiple movement patterns from a single neural strategy. *J. Neurophysiol.* 100, 3394-3406.

Zoubir, A.M., and Boashash, B. (1998). The bootstrap and its application in signal processing. IEEE Signal Processing Magazine 15, 56-76.

Further Supplementary Material: A video is provided, showing several of the test performed with Posturob II on the motion platform in the human posture control laboratory.
