# Supplementary material for: Human-Inspired Eigenmovement Concept Provides Coupling-Free Sensorimotor Control in Humanoid Robot
Source: Front Neurorobot. 2017 Apr 25;11:22. doi: 10.3389/fnbot.2017.00022 (PMC5403929; doi:10.3389/fnbot.2017.00022)
Supplement: Supplementary file 3 [file Appendix2.docx]

**Readme video (PDF**)

Video for Supplementory Materials to

**Human-inspired Eigenmovement concept provides coupling-free sensorimotor control in humanoid robot**

by A.V. Alexandrov, V. Lippi , T. Mergner, А.А. Frolov, G. Hettich, D. Husek

a) This film shows examples of testing the Eigenmovement controller in the robot PostuRob II.

PostuRob II has previously been used in a neurorobotics approach to test a human-derived model of biped balancing during support surface tilt as describe e.g. in [1]. This previous paper lists details of the robots human-like anthropometrics, the actuators (pneumatic artificial muscles, used in a force control actuation involving force sensors in the muscle fixations), the robot’s structure (representing a double inverted pendulum, DIP, mechanics with the feet freely standing on firm support), human-inspired sensors (see below), and the test bed consisting of a motion platform in a human posture control laboratory.

Initially, the film presents a picture with the robot’s sensors. The sensors used in the present experiments are the proprioceptive signals: joint angle and angular velocity of the ankle joints and the hip joints (taking mean value of the right and left sides, respectively), which were used by the Eigenmovement controller described in the paper. (The pressure sensors in the foot soles shown in the picture were not used.) In the last experiment of the film (Fifth Experiment, below), where the robot compensates support surface (SS) tilts, the robot’s equivalent of the vestibular sensor (the IMU) was included into the control. Fusing the IMU signal of sagittal trunk rotation in space with the proprioceptive signal of leg rotation with respect to the trunk, an estimate of the leg’s orientation is space was obtained (see [1]) and was used for balancing.

The following film sequences show PostuRob II controlling stance:

(Experiment 1) Rapid trunk bending (at 1.2 Hz; small leg segment excursions are due to real world imperfections). (Experiment 2) Trunk bending (0.2 Hz) during sinusoidal support surface (SS) translations. (Experiment 3) Balancing during transient SS translations. (Experiment 4) Balancing during pseudorandom SS translations (frequency spectrum see [1]). (Experiment 5) Balancing during sinusoidal SS tilts (0.1 Hz) of 4° peak amplitude.

b) Download Size: < 15 MB;

c) *Player Information*: Video players that run mp4 format;

d) *Packing List*: Readme file, video;

e) *Contact Information*: T. Mergner, Neurocenter, University Clinics, D-79106 Freiburg, Germany. Email: thomas.mergner@uniklinik-freiburg.de

[1] G. Hettich, L. Assländer, A. Gollhofer, T. Mergner, "Human hip–ankle coordination emerging from multisensory feedback control", *Human Movement*

*Science,* Volume 37, pp. 123-146, 2014 ISSN 0167-9457,

http://dx.doi.org/10.1016/j.humov.2014.07.004
